# Characteristics and predictors for tuberculosis related mortality in Denmark from 2009 through 2014: A retrospective cohort study

**DOI:** 10.1371/journal.pone.0231821

**Published:** 2020-06-04

**Authors:** Inge K. Holden, Troels Lillebaek, Peter H. Andersen, Christian Wejse, Isik S. Johansen

**Affiliations:** 1 Department of Infectious Diseases, Odense University Hospital, Odense, Denmark; 2 Mycobacterial Centre for Research Southern Denmark–MyCRESD, Odense, Denmark; 3 International Reference Laboratory of Mycobacteriology, Statens Serum Institut, Copenhagen, Denmark; 4 Department of Infectious Disease Epidemiology and Prevention, Statens Serum Institut, Copenhagen, Denmark; 5 Department of Infectious Diseases, Aarhus University Hospital, Aarhus, Denmark; 6 Department of Clinical Research, University of Southern Denmark, Odense, Denmark; Agencia de Salut Publica de Barcelona, SPAIN

## Abstract

**Objectives:**

Mortality from tuberculosis (TB) has been declining since 2000, nevertheless there is still a significant number of patients who die before or during TB treatment. The aims were to examine and describe predictors associated with TB related mortality.

**Methods:**

Patients notified with TB from 2009 though 2014 in Denmark were included. Data were extracted from national registers and patient records were examined for clinical information and treatment outcome. Cox proportional hazards regression was used to examine TB related mortality.

**Results:**

A total of 2131 cases were identified, 141 (6.6%) patients died before or during TB treatment. TB related mortality accounted for 104 cases (73.8%) and decreased significantly from 6.7% to 3.2% (p = .04) during the study period. Within 1 months of diagnosis, 49% of TB related deaths had occurred. The strongest risk factors present at time of diagnosis, associated with TB related mortality, were: age > 70 years, Charlson comorbidity index > 1, alcohol abuse, weight loss, anemia, and C-reactive protein > 100 mg/L (p < .05).

**Conclusion:**

The majority of TB related deaths occurred soon after diagnosis, emphasizing that TB patients identified to have a high risk of mortality should be closely monitored before and during the intensive treatment period to improve their outcomes.

## Introduction

From 2000 to 2016, the mortality rate of tuberculosis (TB) has been declining with the greatest decline observed in Western Europe. However, globally, TB remains the number one cause of death among infectious diseases in people older than 5 years. The proportion of people with TB who dies varies from less than 5% in Western Europe to more than 20% in Sub-Saharan Africa [1].

Denmark is a low incidence country with a TB incidence of 4.8 pr. 100.000 in 2017 and patients have free access to medical care. Nevertheless, there is still a number of people who die before or while on TB treatment. Earlier Danish studies have demonstrated increased long term mortality in TB patients, and a recent Danish study, found the overall hazard ratio of death was 2.45 during two year follow up [2, 3]. The study identified increasing age and increasing number of comorbidities to be associated with mortality. It was register-based and did not include clinical information. There are no recent Danish studies examining the clinical risk factors associated with mortality before and during TB treatment.

In two recent studies, we reported that 80.5% and 86.7% pulmonary TB (PTB) and extrapulmonary TB (EPTB) cases, respectively, achieved treatment success, and the proportion of patients who died was 7.5% and 3.3%, respectively [4, 5].

In order to improve treatment success rates and potentially avoid death, knowledge regarding predictors associated with unsuccessful treatment outcome and death present at time of diagnosis is valuable. Most studies, which have examined risk factors associated with mortality in TB patients, examine all-cause mortality, rather than TB related mortality [6–12]. However, knowing if the cause of death is related to TB can be valuable when monitoring TB control programs and might help to identify effective interventions.

Also, only few studies report the number of TB patients who are diagnosed postmortem and the proportion varies from 1.7% to 3% [13–15]. Patients diagnosed with TB postmortem possibly represents a source of transmission, identifying these cases can potentially reduce mortality and limit transmission.

The aims of this study were to determine mortality rate by year before and during TB treatment and examine and describe the characteristics of predictors associated with TB related mortality.

## Materials and methods

We included all patients notified with TB in Denmark from January 1^st^, 2009 to December 31^st^, 2014.

### Data source

#### Notification data

TB notification has been mandatory in Denmark since 1905 [16], and for decades, cases have been notified according to WHO definitions [17]. We retrieved the following information from surveillance: identification codes (Civil Registration numbers–CRN), demographics, immigrant status, date of notification, and hospital from which the patient was notified.

Immigrant status was defined as patients born abroad or those born in Denmark for whom one or both parents had been born abroad, including in Greenland.

#### Microbiological data

The International Reference Laboratory of Mycobacteriology (IRLM) at Statens Serum Institut (SSI) preforms mycobacteria diagnostics on all suspected patients in the country. They provided all bacteriologic data for the study.

#### Danish national patient registry

The Danish National Patient Registry (DNPR) contains data on all admissions to Danish public hospitals since 1977 and data on outpatient contacts since 1994 [18].

Data was obtained on all patients, who were notified with TB in Denmark during the study period. Data from DNPR was used to calculate Charlson comorbidity index (CCI) based on the patients discharge diagnoses [19].

#### Data linkage

In order to cross-link the registers, we used the unique Danish CRN, which is assigned to all residents of Denmark at birth or after residing legally in Denmark for 3 months. Patients who do not meet the criteria for obtaining a CRN are assigned a temporary CRN at first point of contact with the healthcare system. To accommodate for alterations in temporary CRN probabilistic linkage was done.

All CRNs obtained from the notification system were linked to the DNPR data and to the data from IRLM.

#### Hospital records

For all patients identified through the notification system, medical records were reviewed for socio-demographics, clinical characteristics and TB treatment information. TB treatment outcome was obtained from hospital records, since national reporting is voluntary and thus data incomplete.

Patients’ delay was calculated as time from TB related symptom onset until first hospital contact, whereas doctors’ delay was calculated as time from first contact to the hospital to start of TB treatment. Diagnostic delay was described as the sum of the patient’s and the doctor’s delay.

TB location was classified according to origin of sample submitted for culture and information from medical records. Cases were divided into 3 categories according to TB localization: PTB if TB was exclusively in the lungs, the tracheobronchial tree or the larynx, EPTB if TB involved organs not included in PTB and finally PTB+EPTB if TB localization was defined in accordance with PTB and EPTB.

The end of the TB treatment initiation phase was defined as treatment duration between one and two months.

Alcohol abuse was quantified according to the Danish Health Authorities recommendations (women > 14 units, men > 21 units of alcohol per week) [20].

Nonadherence was defined as 2 or more non-attendance for clinical appointments and/or if mentioned in patient records.

Patients were excluded if there were diagnosed and treated for latent TB infection or infection with the Bacillus Calmette-Guérin (BCG) strain of *Mycobacterium Bovis* due to intravesical BCG instillation, or if treatment was completed outside of Denmark.

Additional TB cases were included if a relapse/new episode of TB was identified in the patient record. A new episode/relapse was defined according to WHO/ECDC guidelines and cases were only included once during a 12 month period [17, 21].

Mortality data were assigned as TB-related or unrelated, and for TB related death it was further accessed whether TB was the direct cause of death. The following criteria were used: TB direct cause of death in which TB disease directly lead to organ failure or fatal complication, TB related death in which death from another cause had been precipitated by TB disease, TB unrelated death in which the cause of death was independent of TB disease.

### Statistical analysis

All TB cases were included in the data analysis describing patient characteristic. Categorical data was described by total and percentages, the denominator for calculated percentages was the number of cases with known information. Data comparisons were made using the chi-square test or Fisher’s exact test if 20% of expected cell value were ≤5. Continuous variables were described as medians and interquartile ranges and compared using the Wilcoxon rank sum test. A p-value of less than 0.05 (5%) was considered statistically significant.

In the present analysis cases were censored at the end of TB treatment and the outcome: TB related mortality was studied. Cox proportional hazards regression was used to investigate TB related mortality, defined as TB related death before or during TB treatment. Risk factors were defined at baseline. Univariable and multivariable analyses between TB related mortality and potential risk factors were performed.

The multivariable model was built in a forward method, variables for multivariate analysis were selected if they showed a univariate association with TB related mortality (p < .05) and included in the final model if this led to significant improvement.

Proportionality assumption was assessed by using the Schoenfeld and scaled Schoenfeld residuals and the fit of the model was evaluated by using the Cox-Snell residuals.

Survival curves were constructed for 3 risk groups identified on the basis of the multivariable analysis.

### Ethics

The study was approved by the Danish Data Protection Agency (Jnr. 15/34961) and the Danish Health Authority (Jnr 3-3013-1213/1). In accordance with Danish law, observational studies performed in Denmark do not need approval from the Medical Ethics Committee or written consent from participants. All data from patient records and the databases were anonymized when they were received and all analyses are presented anonymously.

## Results

### Population

A total of 2150 TB cases were notified with TB of among whom 36 were excluded and 17 included twice due to a new episode of TB, resulting in 2131 cases in total (S1 Fig). A total of 141 (6.6%) patients died, including 104 (73.8%) who were classified as TB related. Among these, deaths were exclusively attributed to TB in 47 (45.2%) cases. From 2009 to 2014, TB related death decreased significantly from 6.7% to 3.2% (p = .04).

### Socio-demographic and clinical characteristics

The general characteristics of the population are summarized in Table 1. The majority were male (61.5%) and the median age of the population was 42 years (IQR: 29–53). In total, 1059 (49.7%) immigrants, 714 (33.5%) Danes and 358 (16.8%) of Greenlandic origin. The majority of immigrants originated from Asia (47.3%), whereas cases Africa and Europe accounted for 28.8% and 22.9%, respectively.

**Table 1:** TB related mortality^1^ and overall mortality, by sociodemographic risk factors among patients with TB in Denmark 2009–2014.

	Total n (%)	Dead any cause n (%)	p-value	TB-related mortality^*1*^ (n = 104)	p-value
***Sex***			0.001		0.003
*Male*	1311 (61.5)	106 (8.1)		78 (6.1)	
*Female*	820 (38.5)	35 (4.27)		26 (3.2)	
***Age***			0.000		0.000
*0–29*	536 (25.2)	3 (0.6)		3 (0.6)	
*30–49*	882 (41.4)	45 (5.1)		35 (4.0)	
*50–69*	575 (27.0)	59 (10.3)		40 (7.2)	
*≥ 70*	138 (6.5)	34 (24.6)		26 (20.0)	
***Country of origin***			0.000		0.000
*Denmark*	714 (33.5)	86 (12.0)		61 (8.9)	
*Greenland*	358 (16.8)	32 (8.9)		24 (6.7)	
*Immigrant*	1059 (49.7)	23 (2.1)		19 (1.8)	
***Predisposing factors***					
*Alcohol abuse^*2*^*	699 (34.4)	86 (12.3)	0.000	66 (9.7)	0.000
*Tobacco*	1152 (56.7)	97 (8.4)	0.000	71 (6.3)	0.001
*Cannabis*	370 (18.9)	36 (9.7)	0.002	24 (6.7)	0.037
*Illegal drug use within past year*	165 (7.8)	22 (13.3)	0.000	13 (8.3)	0.03
*Homelessness*	215 (10.2)	19 (8.8)	0.15	15 (7.1)	0.109
*History of incarceration*	39 (1.8)	5 (12.8)	0.11	4 (10.5)	0.096
*Charlson comorbidity index*			0.000		0.000
*0*	1398 (68.2)	46 (3.3)		37 (2.7)	
*1*	317 (15.5)	34 (10.7)		25 (8.1)	
*≥2*	334 (16.3)	61 (18.3)		42 (13.3)	
*Previous TB*	304 (14.8)	27 (8.9)	0.13	21 (7.1)	0.106
*HIV positive*	62 (4.2)	5 (8.1)	0.31	4 (6.6)	0.209
*Diabetes*	96 (4.7)	17 (17.7)	0.000	8 (9.2)	0.08
*Cancer*	105 (5.1)	17 (16.2)	0.000	13 (12.9)	0.000
*Moderate/severe renal disease*	47 (2.3)	12 (25.5)	0.000	7 (16.7)	0.001
*History of mental illness*	134 (6.3)	12 (9.0)	0.26	8 (6.2)	0.520

1: TB disease lead directly to organ failure or fatal complication OR death from another cause had been precipitated by TB disease.

2: More than 14 units pr. week of alcohol for women and more than 21 units for men

Danes were significant older and had more co-morbidities when compared to immigrants and Greenlanders (p < .01), whereas significantly more patients originating from Greenland had the following risk factors: homelessness, alcohol abuse, smoked cannabis or tobacco (p < .01).

The localization of TB varied significantly according to gender and immigration status; PTB accounted for 89.2% of the cases among Danes and 95.5% among Greenlanders, while only 66.3% of the immigrant cases had PTB (p < .01).

In total, 62 (4.2%) patients were HIV positive, however only 69.9% of the population had a HIV test done before or during TB treatment. During the study period the proportion of patients who were tested for HIV increased significantly from 49.9% to 84.0%.

### Characteristics of mortality

As shown by Table 2, PTB was the most frequent localization and represented the majority of deaths. However, the mortality rate was highest among patients who were diagnosed with both PTB and EPTB. The percentage of culture positive patients were significantly higher among the deceased, and one patient was diagnosed with multidrug resistant TB.

**Table 2:** TB-related mortality^1^ and overall mortality, by site of TB and diagnostics; Denmark 2009–2014.

	Total n (%)	Dead any cause n (%)	P-value	Tb-related mortality^1^ n (%)	P-value
*PTB^*2*^ only*	1554 (72.9)	111 (7.1)	0.001	81 (5.3)	0.001
*PTB^*2*^ + EPTB^*3*^*	127 (6.0)	15 (11.8)		13 (10.4)	
*EPTB^*3*^ only*	450 (21.1)	15 (3.3)		10 (2.3)	
***Diagnosis supported by:***					
*Microscopy positive*	497 (23.4)	51 (10.3)	0.000	38 (7.9)	0.001
*Culture positive*	1753 (85.8)	136 (7.8)	0.000	102 (5.9)	0.000
*NAA^*4*^ positive*	1107	93 (8.4)	0.01	68 (6.3)	0.019
*Drug susceptible TB*	1554	120 (7.7)		94 (6.2)	
*Isoniazid monoresistant*	93	7 (7.5)	0.94	4 (4.4)	0.509
*MDR^*5*^ TB*	12	1 (8.33)	0.95	0	0.396
*XDR^*6*^ TB*	1	0	0.77	0	0.798

1: TB disease lead directly to organ failure or fatal complication OR death from another cause had been precipitated by TB disease.

2: Pulmonary tuberculosis

3: Extrapulmonary tuberculosis

4: Nucleic Acid Amplification

5: Multidrug resistance tuberculosis

6: Extensively drug-resistant tuberculosis

In total, 28 (19.9%) patients died before treatment was initiated. The diagnosis was culture verified in 27 (96.4%) patients and in the last case diagnose was verified by microscopy only. The patients were significantly older and had more comorbidities than the patients who were diagnosed while alive and able to initiate TB treatment (p < .01). PTB was the most common site representing 22 cases (78.6%) among these 3 patients were diagnosed with miliary TB. Pleural TB accounted for 3 cases and the remaining cases were diagnosed with meningitis, lymph node and gastrointestinal TB. A total of 24 patients were diagnosed postmortem, 5 were diagnosed at autopsy and 4 of these did not have any registered contacts to hospitals prior to death. The proportion of patients diagnosed postmortem did not change significantly during the study period.

**Fig 1:**
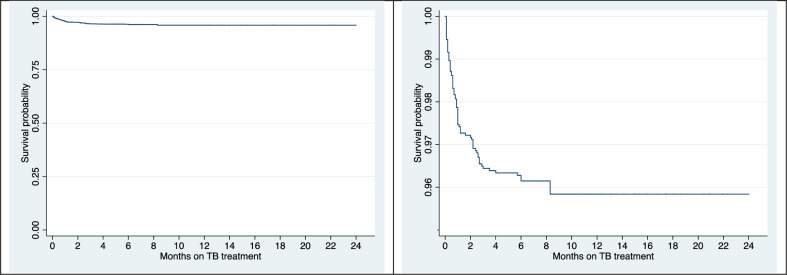
A and B^1^ Survival curve of patients with TB related death^2^ in Denmark 2009–2014. 1: Magnified view of the survival curve 2: The sum of TB related death (death from another cause had been precipitated by TB disease) and death caused by TB (TB disease lead directly to organ failure or fatal complication).

TB related death occurred soon after diagnosis relatively frequently: The Kaplan-Meier survival probabilities were 97.5% (CI: 96.7–98.1%) after the first month of treatment, 96.4% (CI: 95.5–97.2) after 3 months, 96.2% (CI: 95.2–96.9%) after 6 months and 95.8% (CI: 94.8–96.8) after 24 months when treatment was completed for all patients (Fig 1A and 1B).

**Fig 2:**
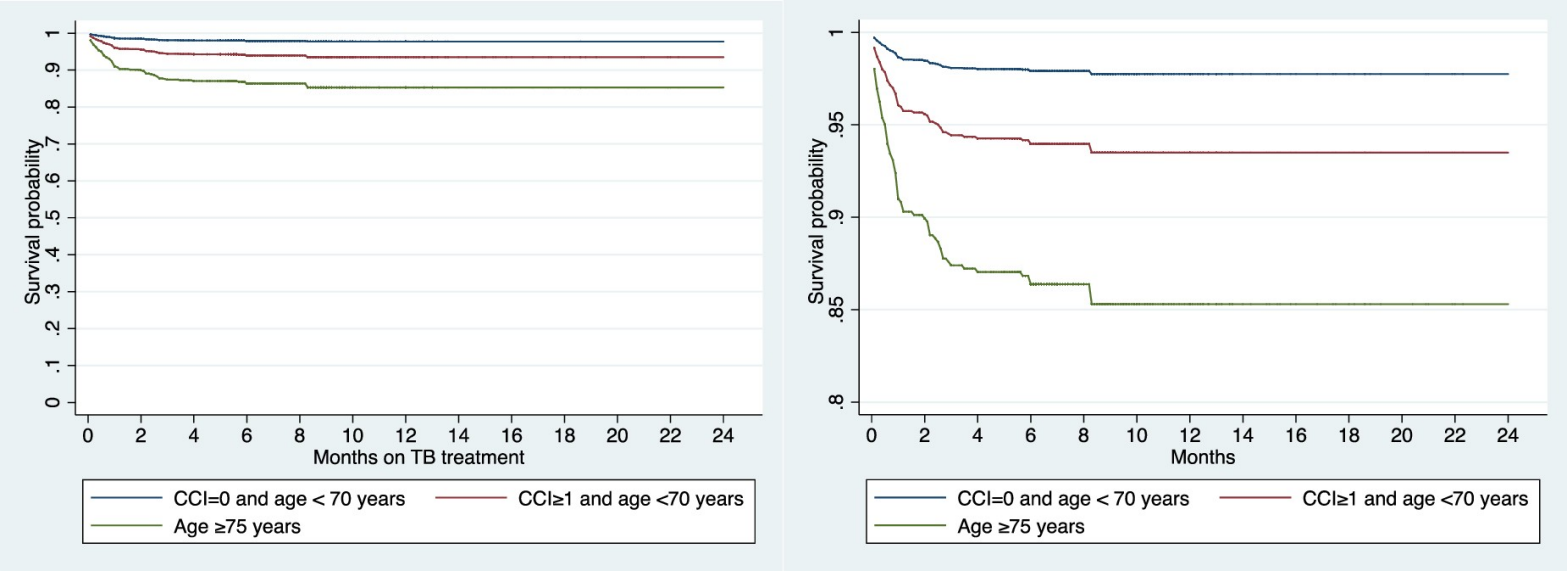
A and B^1^ Survival curves of three risk groups among tuberculosis patients alive at diagnosis. 1: Magnified view of the survival curves CCI: Charlson comorbidity index.

In order to predict outcome of TB patients who were alive and able to start treatment at diagnosis a simple classification was used (Fig 2A and 2B). The Kaplan-Meier probabilities of surviving 3 months after TB treatment initiation was 87.9% (95% CI 80.8–92.5%) for patients above 70 years of age (risk group I), 93.9% (95% CI: 91.6–95.7%) for patients less than 70 years of age and a CCI score ≥ 1 (risk group II), and 98.2% (95% CI: 97.3–98.7%) for patients less than 70 years of age and a CCI score <1 (risk group III). Group I+II represented 756 (35.8%) of the TB cases and accounted 57 (67.1%) of the TB related deaths.

In total 13 patients died towards the end of the initiation phase of these 6 deaths were classified as TB-related. These patients did not differ in terms of age, comorbidity or risk factors. All six TB-related deaths were diagnosed with PTB and two of these had a co-existing EPTB.

Table 3 presents the univariate and multivariable cox regression analysis. After adjusting for age and alcohol use the association between the increased risk of TB related death and country of origin/site of TB/diabetes mellitus disappeared. The final model was fitted on 1608 TB cases, mortality was significantly associated with age above 70 years (p = .001), alcohol abuse (p < .001), CCI ≥2 (p = .01), weight loss (p = .02), anemia (p = .01) and C-reactive protein above 100 mg/L at time of diagnosis (p < .001).

**Table 3:** Risk factors for tuberculosis related mortality, Denmark 2009–2014.

Characteristics at time of TB diagnosis	Factors associated with TB related mortality^*6*^, crude		Factors associated with TB related mortality^*6*^, adjusted	
	HR	[95% CI]	HR	[95% CI]
***Sex***				
*Male*	2.10	1.24–3.56		
***Age (years)***				
*0–29*	RF		RF	
*30–49*	7.41	1.75–31.35	2.17	0.48–9.82
*50–69*	16.19	3.89–67.39	2.82	0.61–13.03
*≥ 70*	37.91	8.76–164.10	13.11	2.86–60.18
***Country of origin***				
*Denmark*	4.09	2.31–7.25		
*Greenland*	3.46	1.76–6.78		
*Immigrant*	RF			
***Predisposing factors***				
*Alcohol abuse^*1*^*	4.43	2.71–7.24	3.57	1.92–6.63
*Tobacco*	2.22	1.32–3.75		
*Cannabis*	1.77	1.04–3.02		
*History of Illegal drug use*	1.65	0.82–3.32		
*Homelessness*	1.54	0.82–2.90		
*History of incarceration*	1.52	0.38–6.14		
*Charlson comorbidity score*				
*0*	RF		RF	
*1*	2.99	1.70–5.25	1.72	0.88–3.34
*≥2*	4.09	2.43–6.87	2.47	1.30–4.69
*HIV*	1.60	0.50–5.13		
*Diabetes*	2.43	1.17–5.07		
*Moderate/severe renal disease*	0.63	0.09–4.46		
*Cancer*	2.25	1.09–4.65		
*History of mental illness*	1.05	0.42–2.62		
***Site of disease***				
*PTB^*2*^ only*	3.00	1.30–6.90		
*PTB + EPTB^*3*^*	6.17	2.22–17.15		
*EPTB only*	RF			
*Identification by Contact Tracing*	0.63	0.27–1.45		
***Clinical symptoms***				
*Patients’ delay > 30 days*	0.94	0.52–1.70		
*Doctors’ delay > 30 days*	0.79	0.46–1.36		
*Diagnostic delay > 60 days*	0.87	0.48–1.58		
*Weight loss*	4.19	2.25–7.82	2.34	1.14–4.81
*Fever*	2.13	1.34–3.40		
*Night sweet*	0.31	0.15–0.64		
***Diagnosed supported by***				
*Microscopy positive*	1.69	1.05–2.71		
*Culture Positive*	13.00	1.81–93.26		
***Lab results***				
*Anemia^*3*^*	5.39	2.62–11.08	2.49	1.22–5.09
*White Blood Cell count*				
*<3.5 10^9^/L*	5.79	1.98–16.92		
*3.5–9 10^9^/L*	RF			
*>9 10^9^/L*	2.97	1.79–4.92		
*C-reactive protein >100 mg/L*	4.24	2.63–6.84	2.67	1.56–4.59
*Adherence^*5*^*				
*Non-adherence*	0.63	0.31–1.28		

Multivariable model fitted on 1608

1. More than 14 units pr. week of alcohol for women and more than 21 units for men

2. Pulmonary tuberculosis

3. Extrapulmonary tuberculosis

4. Women: Hgb<7.5mmol/L. Men: Hgb<8.0mmol/L

5. Non-adherence: if described in patient records and/or ≥ two episodes of non-attendance for clinical appointments

6. TB disease lead directly to organ failure or fatal complication OR death from another cause had been precipitated by TB disease.

### Cause specific mortality

Table 4 summarizes the cause of death divided by TB related death, TB direct cause of death, and TB not related to death. Respiratory failure was the most frequent cause of death overall (n = 59; 41.8%). Cases diagnosed with PTB represented the majority of deaths caused by respiratory failure (n = 51; 86.4%). Multiorgan failure was the second most common cause of death representing 23 cases (16.3%) of these 12 cases (52.2%) were caused by sepsis. TB related death constituted the majority of these cases (n = 16).

In the 7 cases where liver cirrhosis was the cause of death, alcohol was the underlying cause.

**Table 4:** Cause of death divided by: TB related death, TB direct cause of death and death not related to TB.

	Tuberculosis direct cause of death^*1*^			Tuberculosis related death^*2*^			Death not related to tuberculosis^*3*^			
	PTB n (%)	PTB+ EPTB n (%)	EPTB n (%)	PTB n (%)	PTB+ EPTB n (%)	EPTB n (%)	PTB n (%)	PTB+ EPTB n (%)	EPTB n (%)	Total n (%)
*Cause of death*										
*Respiratory failure*	32 (84.2)	3 (8.3)	1 (33.3)	11 (25.6)	1 (14.3)	2 (28.5)	8 (26.7)	1 (50.0)	0	59 (41.8)
*Multiorgan failure caused by sepsis*	0	1 (16.7)	0	7 (16.3)	1 (14.3)	1 (14.3)	2 (6.7)	0	0	12 (8.5)
*Multiorgan failure*	0	0	0	5 (11.6)	1 (14.3)	1 (14.3)	3 (10.0)	0	1 (20.0)	11 (7.8)
*Cardiac disease*	0	1 (16.7)	0	2 (4.7)	1 (14.3)	0	3 (10.0)	0	1 (20.0)	8 (5.7)
*Stroke*	0	1 (16.7)	1 (33.3)	0	1 (14.3)	0	4 (13.2)	0	0	7 (5.0)
*Liver cirrhosis*	1 (2.6)	0	0	0	1 (14.3)	3 (42.9)	2 (6.7)	0	0	7 (5.0)
*Cancer*	1 (2.6)	0	0	3 (7.0)	0	0	0	0	1 (20.0)	5 (3.6)
*Drug overdose*	0	0	0	1 (2.3)	0	0	0	0	1 (20.0)	2 (1.4)
*Haemoptysis*	0	0	0	1 (2.3)	0	0	0	0	0	1 (0.7)
*Unknown*	4 (10.5)	0	1 (33.3)	13 (30.2)	1 (14.3)	0	8 (26.7)	1 (50.0)	1 (20.0)	29 (20.6)
*Total*	38 (80.8)	6 (12.8)	3 (6.4)	43 (75.4)	7 (12.3)	7 (12.3)	30 (81.1)	2 (5.4)	5 (13.5)	141

1: TB disease lead directly to organ failure or fatal complication.

2: Death from another cause had been precipitated by TB disease.

3: The cause of death was independent of TB disease.

## Discussion

This study provides comprehensive information of TB related death and risk factors in Denmark. Despite the high-resource TB low incidence setting, with free and equal access to healthcare, almost 7% of patients diagnosed with TB died and the majority of deaths were related to TB. However, TB related deaths did decrease significantly from 6.7% to 3.2% (p = .04) during the study period. The strongest risk factors present at time of diagnosis and associated with TB related mortality were age > 70 years, CCI ≥ 2, alcohol abuse, weight loss, anemia, and C-reactive protein > 100 mg/L at time of diagnosis (p < .05).

ECDC has reported a 59% decline in TB mortality in Europe between 2008 and 2017 [22]. Likewise, we observed a decline in TB related mortality from 6.75% to 3.2% during the study period, the greatest decline was among Danish cases (6.5% to 2.3%). In consensus with this we observed a decline in the proportion of Danish cases above 65 years (26.3% to 18.8%).

The majority of TB related deaths occurred soon after diagnosis. One in four cases died before treatment initiation and 98% of all deaths had occurred within 6 months of diagnosis. As a result, the duration of different treatment regimens is unlikely to have impacted the statistics. The fact that 49.0% of the patients died within one months of diagnosis makes it questionable if these deaths were preventable, in addition delay in diagnosis was not significantly associated with TB related mortality. However, the most frequent cause of death among the TB related deaths diagnosed antemortem was respiratory failure (51.8%) followed by multiple organ failure (17.7%). Some of these deaths might be preventable and highlights that TB patients should be monitored closely, particularly in the beginning of their TB treatment.

The proportion of patients diagnosed postmortem remained stable and accounted for 15% of deaths, 5 of these patients were diagnosed at autopsy. The annual number of autopsies in Denmark are low and according to The Danish Cause of Death Register, the number of autopsies has been declining in Denmark since 2009 and in 2017 only 2.8% of all deaths resulted in autopsy. This suggests that the number of patients with undiagnosed TB might be greater. The majority of the patients identified postmortem were diagnosed by positive culture from the lung parenchyma (57.1%), insinuating that these patients represent an unidentified source of transmission. The patients were significantly older and had more comorbidities, which can result in difficulties to diagnose as TB can present atypically in the ageing population [23]. This emphasizes that the suspicion of TB should be high among physicians treating older patients.

Age above 70 years was significantly associated with mortality. Earlier studies have indicated that the effects of advanced age can cause more advanced disease, furthermore there is an increased incidence of adverse drug reactions, all contributing to a higher risk of mortality [23].

A significant amount of TB in the older population might be caused by reactivation of latent TB acquired in young age when the incidence of TB in Denmark was significantly higher. There is a higher probability of reactivating TB among the ageing population as advanced age can result in a compromised immune system [24]. In addition, there is a higher prevalence of comorbidities such as cancers, diabetes mellitus and malnutrition which contributes to an increased risk of reactivation of latent TB [25]. Hence, this group of patients will not be identified through contact tracing.

C-reactive protein above 100 mg/L, anemia and weight loss at time of diagnosis were all associated with TB related mortality, this suggests prolonged and more advanced TB disease at time of diagnosis. However, it is important to point out that 13% of the patients had co-excising cancer, which also can result in elevated C-reactive protein, anemia and weight loss, nevertheless the association did persist after adjusting for CCI.

Alcohol abuse was associated with TB related mortality, which is in consistence with earlier studies [7, 9, 14, 26–28]. This is not unexpected as earlier studies have found alcohol abuse to be associated with non-adherence to TB treatment and unsuccessful TB treatment outcome [4, 29–31]. In addition, alcohol abuse has been linked to a compromised immune system and consequently a higher risk of developing more severe forms of TB [32]. All these factors can potentially contribute to a higher risk of mortality in patients suffering from alcohol abuse.

It is well documented that patients with multidrug resistance (MDR) / extensively drug-resistant (XDR) TB have an increased risk of death during treatment [12, 21, 33, 34]. We were not able to demonstrate this association, only one patient with MDR-TB died during treatment; hence patients with drug resistant TB did not contribute significantly to the mortality rate, which is in line with an earlier Danish study [35]. However, the number of patients with MDR/XDR TB was very limited (n = 13). Similarly, the HIV prevalence in Denmark is low (approximately 0.1%) [36], accordingly the number of patients co-infected with HIV was limited, hence the association between HIV and mortality might be underestimated.

The limitations of this study are that the population was identified by notification data, hence patients who are not notified were not included. However, a recent study from Denmark has assessed the underreporting of TB to 7.5%. The non-notified cases were all culture-negative and did not differ significantly in treatment outcome and risk factors from the notified cases [37]. All clinical information was from hospital records; hence no direct patient contact and the quantity and quality of information solely depends on the hospital records. EPTB was not subdivided into types of EPTB due to the limited case number. As a result, we were not able to describe if mortality was associated with specific types of EPTB. In 23.4% of cases, information regarding diagnostic delay was missing, potentially underestimating the effect on TB related mortality. The cause of death was based on information from medical journals, as we did not have autopsy reports, which are often considered the “gold standard” for underlying cause of death. Risk factors associated with TB related death towards the end of the initiation phase might have been underestimated as only 6 patients were classified as TB related death.

CCI could not be assessed for patients with temporary CRN, because they are not registered in the DNPR, these patients accounted for 3.9% (n = 82) of the entire population.

While the study period is from 2009 through 2014, the results are current as TB mortality rates have remained stable to date according to the Danish National TB Surveillance.

## Conclusion

Several risk factors associated with TB related mortality present at time of diagnosis were identified in this study. The majority of deaths occurred soon after diagnosis, emphasizing that patients with TB identified as high risk of mortality should be closely monitored before and during treatment to improve their outcomes.

Furthermore, a significant number of patients were diagnosed postmortem, these patients were older and represented an unidentified source of transmission. An increased focus on TB in the ageing population is recommended.

## Supporting information

S1 Fig Study population. A new episode/relapse was defined according to WHO/ECDC guidelines and cases were only included once during a 12 months period.
(PDF)
