# Supplementary material for: Characteristics and predictors for tuberculosis related mortality in Denmark from 2009 through 2014: A retrospective cohort study
Source: PLoS One. 2020 Jun 4;15(6):e0231821. doi: 10.1371/journal.pone.0231821 (PMC7272085; doi:10.1371/journal.pone.0231821)
Supplement: S1 Fig — A new episode/relapse was defined according to WHO/ECDC guidelines and cases were only included once during a 12 months period. (PDF) [file pone.0231821.s001.pdf]

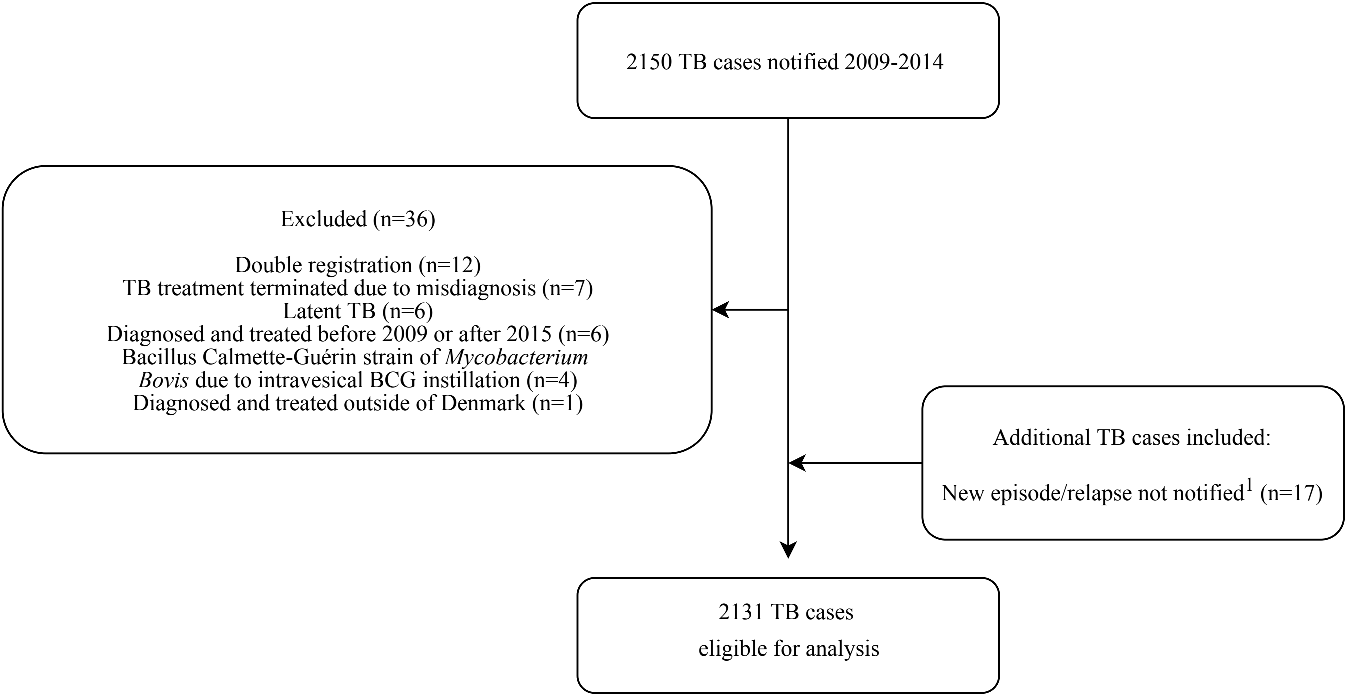
2150 TB cases notified 2009-2014
Excluded (n=36)
Double registration (n=12)
TB treatment terminated due to misdiagnosis (n=7)
Latent TB (n=6)
Diagnosed and treated before 2009 or after 2015 (n=6)
Bacillus Calmette-Guérin strain of *Mycobacterium Bovis* due to intravesical BCG instillation (n=4)
Diagnosed and treated outside of Denmark (n=1)
Additional TB cases included:
New episode/relapse not notified[1] (n=17)
2131 TB cases
eligible for analysis
